# Different Water Use Strategies of Juvenile and Adult *Caragana intermedia* Plantations in the Gonghe Basin, Tibet Plateau

**DOI:** 10.1371/journal.pone.0045902

**Published:** 2012-09-21

**Authors:** Zhiqing Jia, Yajuan Zhu, Liying Liu

**Affiliations:** 1 Institute of Desertification Studies, Chinese Academy of Forestry, Beijing, PR China; 2 Qinghai Gonghe Desert Ecosystem Research Station, Shazhuyu Town, Gonghe County, Qinghai Province, PR China; RIKEN Plant Science Center, Japan

## Abstract

**Background:**

In a semi-arid ecosystem, water is one of the most important factors that affect vegetation dynamics, such as shrub plantation. A water use strategy, including the main water source that a plant species utilizes and water use efficiency (WUE), plays an important role in plant survival and growth. The water use strategy of a shrub is one of the key factors in the evaluation of stability and sustainability of a plantation.

**Methodology/Principal Findings:**

*Caragana intermedia* is a dominant shrub of sand-binding plantations on sand dunes in the Gonghe Basin in northeastern Tibet Plateau. Understanding the water use strategy of a shrub plantation can be used to evaluate its sustainability and long-term stability. We hypothesized that *C. intermedia* uses mainly deep soil water and its WUE increases with plantation age. Stable isotopes of hydrogen and oxygen were used to determine the main water source and leaf carbon isotope discrimination was used to estimate long-term WUE. The root system was investigated to determine the depth of the main distribution. The results showed that a 5-year-old *C. intermedia* plantation used soil water mainly at a depth of 0–30 cm, which was coincident with the distribution of its fine roots. However, 9- or 25-year-old *C. intermedia* plantations used mainly 0–50 cm soil depth water and the fine root system was distributed primarily at soil depths of 0–50 cm and 0–60 cm, respectively. These sources of soil water are recharged directly by rainfall. Moreover, the long-term WUE of adult plantations was greater than that of juvenile plantations.

**Conclusions:**

The *C. intermedia* plantation can change its water use strategy over time as an adaptation to a semi-arid environment, including increasing the depth of soil water used for root growth, and increasing long-term WUE.

## Introduction

Shrubs are a dominant ecotype and are very important in the ecological function of arid and semi-arid ecosystems. Water is a restrictive factor for plant growth because rainfall in these areas is low (mean annual precipitation <500 mm) and unpredictable. The ability to acquire water at shallow depths of soil (recharged by rain), or from deeper soil (recharged by groundwater), and suitable water use efficiency (WUE) are important for the survival, growth and reproduction of shrubs. The strategy of a shrub for water use has been studied by stable isotope technology in many arid and semi-arid zones, including southwestern USA [1]–[7], Mongolia [8] and northern China [9]–[14]. There is generally no stable isotope fractionation during water uptake by roots or transportation of water in the xylem of most species. Thus, the main water source (e.g. rain, snow, river, soil water or ground water) can be distinguished by comparing the δD or δ^18^O ratio of xylem water with that of the potential source water [1], [15]. Further, the δ^13^C ratio of leaves is closely related to the long-term WUE of C_3_ species [15]. However, there is still little knowledge about the strategy for water use by a shrub in different stages of its life history, such as the difference between juvenile and adult plants. Moreover, there are few reports about the effect of plant age on WUE. For example, smaller juveniles of *Chrysothamnus nauseosus* had lower WUE than larger, adult plants as estimated by carbon isotope discrimination in the Great Basin [16]. This might be because it is difficult to confirm the age of shrubs in a natural habitat as, unlike tree species, these perennial plants do not have growth rings.

Natural shrubland and shrub plantations, including *Caragana* spp., form a defense for an oasis to alleviate the damage caused by sandstorms; e.g. in the Gonghe Basin on the northeast Tibet Plateau in Qinghai Province. wherein this area, desertification has occurred during the last century. The environment is semi-arid steppe, shrubland and sandland. In order to defend against damage caused by mobile sand dunes and sandstorms, large areas of trees (e.g. poplar) and shrubs (e.g. willow, legume, salt cedar and sea buckthorn) have been planted inside and outside oases since the 1980s. The shelter belt system was formed to protect farms and villages [17]. However, many shrublands have degraded because of wind erosion, sand deposition, over-grazing and land reclamation. The fixation of mobile sand dunes and the restoration of desertified steppe includes fencing and planting artificial shrubs on reclaimed land. Therefore, the water use strategy of a shrub plantation needs to be understood to increase our knowledge of their ecophysiology, especially water use in adapting to a semi-arid environment, and to evaluate its sustainability and long-term stability. We hypothesize that shrubs use mainly deep soil water and WUE increases with time on sand dunes.

## Results

### Precipitation at Gonghe Station before the survey in 2009

**Figure 1:**
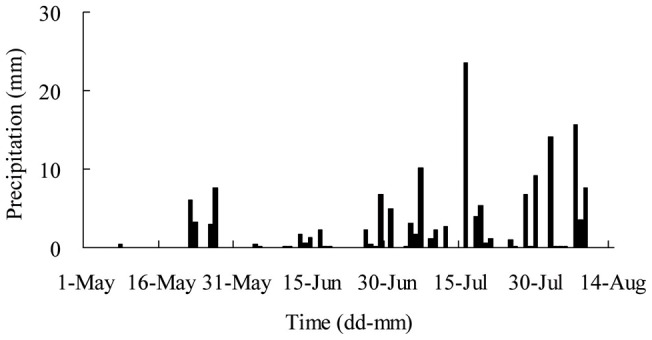
Daily precipitation before field survey (from May 1 to August 10) in 2009 at Qinghai Gonghe Desert Ecosystem Research Station.

Before the field survey at the Gonghe Station in mid-August 2009, the total precipitation of the growing season (May 1–August 13) in 2009 was 155.5 mm. The precipitation in May, June and July was 20.2, 16.1 and 78.0 mm, respectively. The maximal daily precipitation (23.5 mm) occurred on July 16. The total precipitation from 1–9 August was 41.2 mm. Three rainfall occurred during the first ten days in August: August 2, 8.3 mm; August 7, 8.5 mm; and August 9, 3.5 mm (Fig. 1).

### Soil water content of sand dunes

**Figure 2:**
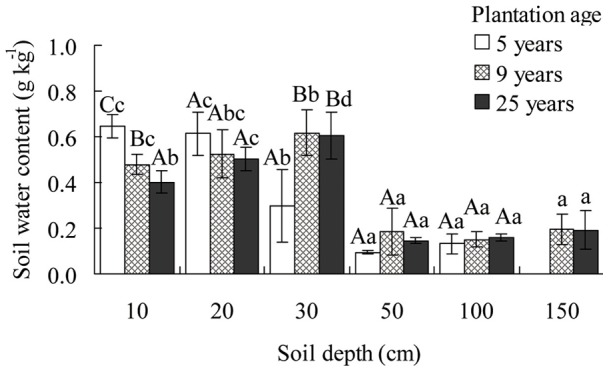
Soil water content (mean ± SE) of sand dunes in three different ages of *Caragana intermedia* plantations. Four replicates of soil sample was take by soil auger in each age of *C. intermedi*a plantations. Different lowercase letters indicate significant difference in water content at different soil depths, and different uppercase letters indicate significant difference in water content in different plantation ages, according to Duncan's test (*p*<0.05).

In the middle of August, soil water content was affected significantly (*p*<0.05) by soil depth in the three *C. intermedia* plantations of different ages. The water content in shallow soil (depth 10–30 cm) was significantly higher (*p*<0.05) than that in soil at depths ≥50 cm. Soil water content was affected significantly by plantation age in 10 cm (*p*<0.001) or 30 cm (*p*<0.01). The water content of 5 years *C. intermedia* plantation was significantly higher (*p*<0.05) than that of 9 years or 25 years plantation in 10 cm. However, the water content of 5 years *C. intermedia* plantation was significantly lower (*p*<0.05) than that of 9 years or 25 years plantation in 30 cm. Soil water content was 0.3–0.6 g kg^–1^ at a soil depth of 10 cm, 0.4–0.6 g kg^–1^ at 20–30 cm; and <0.2 g kg^–1^ at a soil depth of ≥50 cm (Fig. 2).

### The δD and δ^18^O ratio of water in *C. intermedia* xylem, rain, well and soil

**Figure 3:**
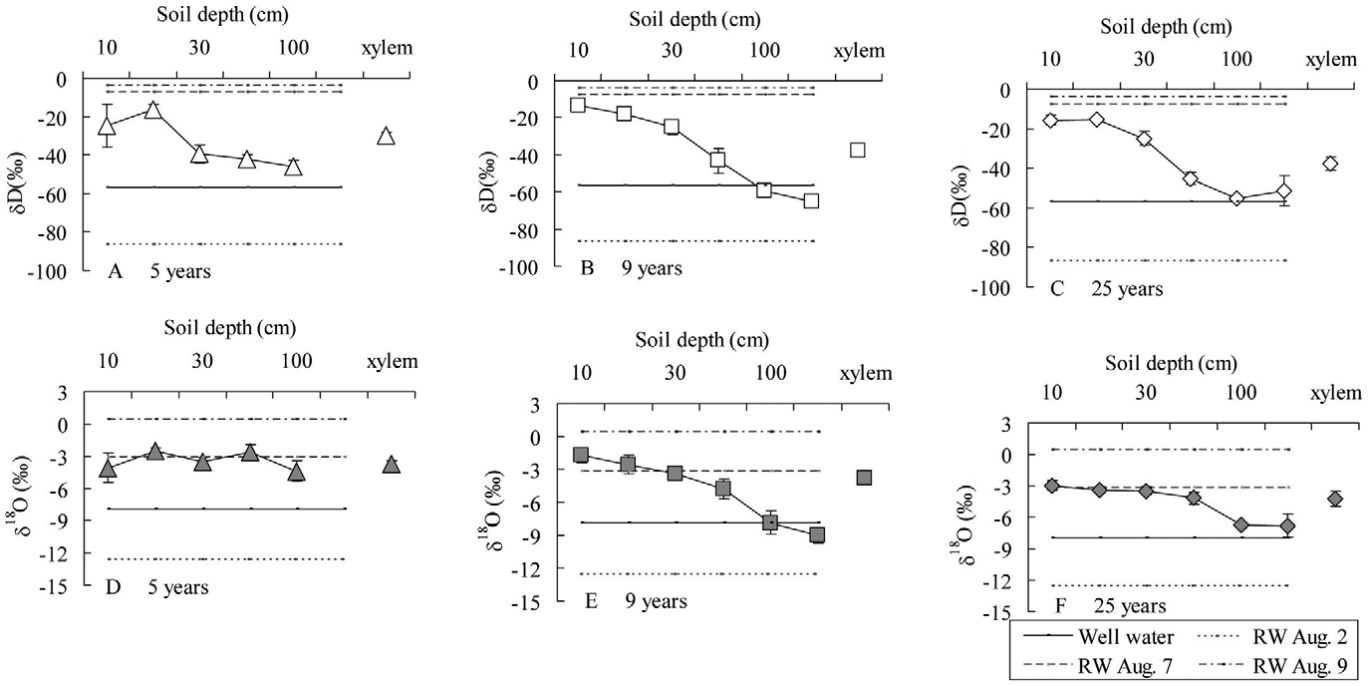
The δD and δ^18^O ratio of *Caragana intermedia* xylem water, different depths of soil water and rain water (mean ± SE). RW means rain water. Four replicates of xylem or soil sample was take in each age of *C. intermedia* plantations. Three replicates of rain water was take after each rainfall.

The δD ratio of xylem water in 5 years old *C. intermedia* was close to that of soil water at depths of 20–50 cm (Fig. 3A). The δD ratio of xylem water in 9 and 25 years old *C. intermedia* was similar to those of soil water at depths of 30–50 cm (Fig. 3B, C). The δD ratio of these soil water samples was similar to that of rainwater August 7 (8.5 mm). Moreover, δD ratio of 100 cm and 150 cm soil water in the 9 and 25 years old *C. intermedia* plantation was similar to that of well water (Fig. 3B, C). By contrast, the δ^18^O ratio of xylem water in 5, 9 and 25 years old *C. intermedia* was close to that of soil water at a depth of 10–50 cm (Fig. 3D, E, F). The δ^18^O ratio of these soil water samples was similar to that of rainwater in August 7 (8.5 mm). Moreover, δ^18^O ratio of soil water at depths of 100 and 150 cm in the 9 and 25 years old *C. intermedia* plantation was similar to that of well water (Fig. 3E, F).

Iso-Source analysis showed the 5 years old *C. intermedia* plantation used mainly 10–30 cm depth soil water, which accounted for 73.5% of its total water source. However, the 9 or 25 years old *C. intermedia* plantations used mainly soil water at a depth of 10–50 cm, which accounted for 82.1% and 87.9% of the total water source, respectively (Table 1).

**Table 1:** Water use ratio (mean ± SD) of different source for three ages of *Caragana intermedia* plantations.

Plantation age	5 years	9 years	25 years
10 cm soil	0.301±0.168	0.267±0.158	0.105±0.074
20 cm soil	0.317±0.141	0.236±0.174	0.082±0.064
30 cm soil	0.117±0.088	0.195±0.165	0.140±0.103
50 cm soil	0.085±0.088	0.123±0.106	0.552±0.041
100 cm soil	0.102±0.080	0.064±0.054	0.049±0.038
150 cm soil	/	0.053±0.045	0.043±0.034
Well water	0.077±0.053	0.063±0.054	0.030±0.025

### Root density of *C. intermedia* plantations of different ages

**Figure 4:**
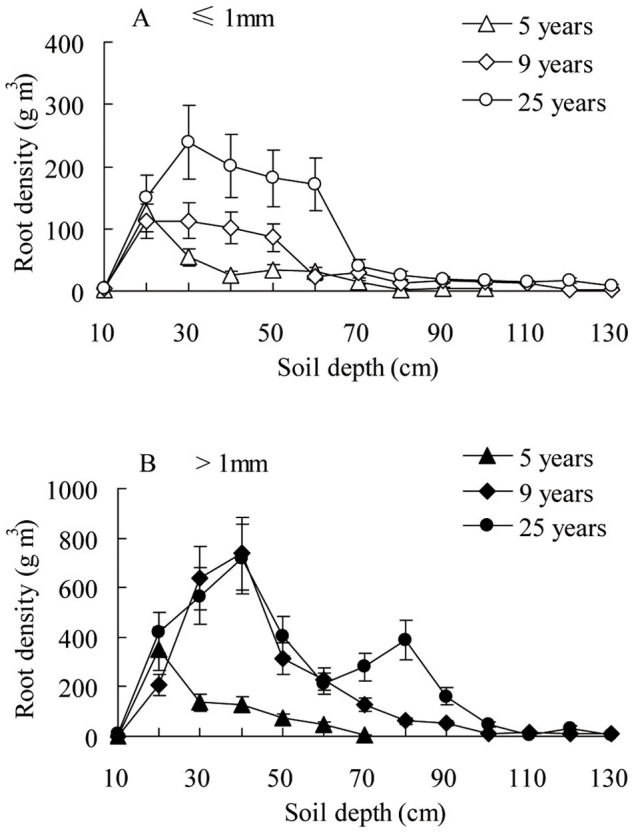
Root density of different ages of *Caragana intermedia* plantations. Three replicates of soil profile (50 cm ×50 cm) was dug in each age of *C. intermedia* plantation.

The fine roots (diameter ≤1 mm) of *C. intermedia* were distributed mainly in sub-surface soil. The distribution range of 5, 9 and 25 years old *C. intermedia* were 20–30, 20–50 and 20–60 cm, respectively. The maximal depth at which the fine roots were distributed widely in soil increased with time, from 30 cm in 5 years, to 50 cm in 9 years and to 60 cm in 25 years (Fig. 4A). The prop roots (diameter >1 mm) were distributed in the sub-surface and in deep soil. The distribution range of prop roots for of 5, 9 and 25 years old *C. intermedia* were 10–40, 10–60 and 10–80 cm, respectively (Fig. 4B).

### Leaf carbon isotope discrimination of *C. intermedia* plantations of different ages

**Figure 5:**
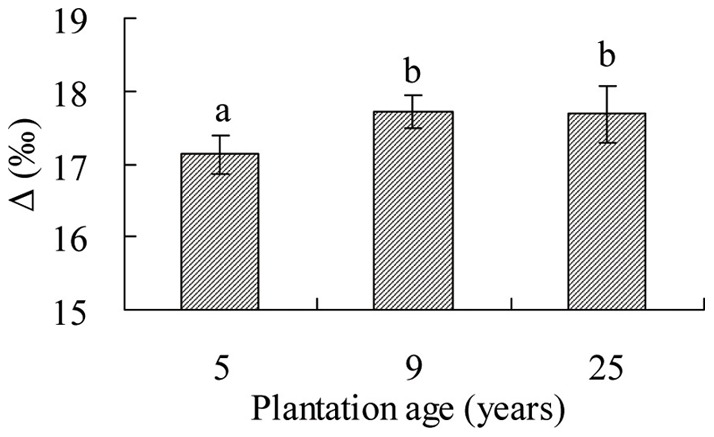
Leaf carbon isotope discrimination of different ages of *Caragana intermedia* plantations. Four replicates of mature leave sample was take in each age of *C. intermedia* plantations.

The effect of age on carbon isotope discrimination (Δ) of *C. intermedia* leaves was significant in the Gonghe Basin (*p*<0.05). The Δ ratio of *C. intermedia* leaves in 9 and 25 years old plantations was significantly higher (*p*<0.05) than that in the 5 years old plantation (Fig. 5).

**Table 2:** Community characteristics in three ages of *Caragana intermedia* plantations.

Plantation age	5 years	9 years	25 years
Longitude	E 100°13′55′′	E100°13′56′′	E 100°16′
Latitude	N 36°14′54′′	N36°15′05′′	N 36°17′19′′
Altitude (m)	2890	2870	2878
Habitat	semi-fixed dune	fixed dune	fixed dune
Height (m)	0.55±0.11	1.24±0.21	1.65±0.19
Crown diameter (m × m)	0.8×0.8	1.08×1.10	2.99×3.47
Total coverage (%)	55	65	80

## Discussion

### Main water source of *C. intermedia* plantations of three different ages

Soil water is one of the most important sources that shrubs can use during the growing season in arid and semi-arid zones. This study showed that during the different stages of its life cycle, *C. intermedia* uses soil water from different depths. It uses shallow soil water (10–30 cm) in juvenile plants (5 years old) and deeper soil water (10–50 cm) in mature plants (9 and 25 years old) (Table 1, Fig. 3). Because soil water in surface (10–30 cm) is abundant than deeper depth (50–150 cm) (Fig. 2), *C. intermedia* mainly uses upper soil water based on its availability. The levels of stable isotopes of hydrogen and oxygen indicate soil water at these depths is recharged by summer rain (Fig. 3). Therefore, summer rain is the most important water source for the survival of *C. intermedia* in Gonghe Basin. Earlier studies found similar phenomena in desert shrubs. *Haloxonlon ammodendron* plantations on sand dunes at the southeastern edge of the Badain Jara Desert depend primarily on deep soil water, and some groundwater in summer. The main depth of soil water use increases with time: from 50–200 cm in a 2 years old plantation, to 100–200 cm in a 5 years old plantation and 150–200 cm in a 10 years old plantation [14]. The δ^18^O ratios indicate young seedlings and older *Artemisia rothrockii* plants use deeper water than most herbs on the montane meadows of the Sierra Nevada Mountains [18].

Many shrubs use different depths of soil water in steppes, sandlands and deserts. For example, the co-dominant shrub *Atriplex canescens* uses mainly water from spring and summer precipitation extracted from subsurface soil layers on Colorado [2]. However, four dominant shrubs (*Artemisia filifolia*, *Coleogyne rammosissima*, *Quercus havardii* and *Ephedra viridis*) take up water from the shallow soil layers [3], [4]. Moreover, from 2–20 mm, with the increase of water by deuterium-labeled irrigation, two shrubs (*Ceratoides lanata* and *Gutierrezia sarothrae*) were found to use more water in spring and summer [5]. *Chrysothamnus greenei* uses only soil water that is recharged by rain. Meanwhile, *Sarcobatus vermiculatus* and *Chrysothamnus nauseosus* use soil water at a depth of 0.3–0.4 m that is recharged by rain during the monsoon season in a wet year but from deep soil and groundwater in drier years [6]. The sub-shrub *G. sarothrae* and *Ceratoides lanata* take up deeper soil water under drought conditions and shallow soil water after a large rainfall in summer. However, only *C. lanata* continues to take up soil water and *G. sarothrae* is dormant after a particularly dry summer [7]. In the montane larch forest of northeastern Mongolia, the cinquefoil shrub *Potentilla fruticosa* uses shallow soil water in response to recent rainfall in July and August [8]. In the Mu-Us Sandland of northern China, the evergreen shrub *Sabina vulgaris* uses relatively deep soil water (50–150 cm) as well as groundwater. However, the deciduous shrub *Artemisia ordosica* uses only shallow soil water (<50 cm) [9]. *A. ordosica* takes advantage of deeper soil water that is recharged by large rainfall (65.3 mm) [10]. In the northeastern Ulanbuh Desert, *Caragana korshinskii* uses mainly deep soil water (60–90 cm) as well as surface soil water (0–30 cm) in late autumn. However, *Nitraria tantutorum* always uses sub-surface soil water (30–60 cm) [12]. At the southeastern edge of the Badain Jaran Desert, both *N. tangutorum* and *Artemisia arenaria* growing on interdunes use sub-surface (30–90 cm) soil water in May. These two shrubs use surface soil water (0–30 cm) in July, which was recharged by a little rainfall in spring and early summer. However, they use deep soil water (>120 cm) in September, which is recharged by rainstorms in late summer [13]. In general, it is suggested that plants use more soil water recharged by summer rain in regions receiving a substantial part of the annual precipitation in the summer [6]. Moreover, shrubs can use soil water that is recharged by other routes. For example, measurement of hydrogen stable isotope indicates *Caragana microphylla* exhibits deep soil water that is recharged by winter snow on the steppe of Inner Mongolia [11].

The difference in soil water use among these shrubs might be related to their different root distributions. Deep-rooted shrubs use mainly deep soil water or ground water. On the other side, shallow-rooted shrubs use only shallow soil water. Moreover, shrubs with dimorphic roots can use both shallow and deep soil water. The results of our field survey indicate that both fine roots and prop roots of *C. intermedia* are distributed mainly in surface soil (within 50 cm) (Fig. 4). Therefore, the root distribution pattern allows *C. intermedia* to use surface soil water recharged by rain. Moreover, the majority of fine roots penetrate into deeper soil with time. Therefore, *C. intermedia* can use water from deeper soil after it grows up. A similar phenomenon has been reported for other work. For example, in the Mu Us sandland, the root density of *A. ordosica* is maximal near the surface and decreases rapidly by a depth of 50 cm, with very few roots near the groundwater table (1.1 m). In contrast, *S. vulgaris* roots are more consistent until above the groundwater table (1.2 m). Therefore, the root system of *S. vulgaris* is able to use soil water at many depths. However, the roots of *A. ordosica* are structured for consumption of shallow soil water [9]. The fine root area density of *Caragana korshinskii* declined with increased soil depth, with 96.6% of all fine roots concentrated in the upper 1 m layer of sandy soil in the semi-arid region of the Loess Plateau [19].

### Water use efficiency in *C. intermedia* plantations of three ages

The long-term WUE of *C. intermedia* decreases with time, as indicated by carbon isotope discrimination (Δ) in the leaf. The WUE of plantations of adult *C. intermedia* (9 and 25 years old) was lower than that of juvenile plants (5 years old) (Fig. 5). The effect of plant age on leaf Δ has been reported for other species, such as the herbaceous desert perennial *Cryptantha flava*. It might be due to older plants maintaining a higher photosynthetic rate but a stomotal conductance similar to that of younger plants late in the growing season [20]. An earlier study indicated that other factors have effects on WUE as estimated by the Δ ratio. For example, WUE in a single plantation of 4 years old *C. korshinskii* is lower than that of a plantation mixed with *Artemisia ordosica* on the southeastern margin of the Tengger Desert. This might be correlated with less soil evaporation of water from the soil surface owing to the good canopy coverage provided by *A. ordosica* [21].

### Conclusions

In conclusion, the main water source of *C. intermedia* plantations of different ages is affected by their root distribution (especially that of the fine roots). This legume shrub use shallow soil water in juvenile plants and deeper soil water in adult plants. The long-term WUE of plantations of adult *C. intermedia* is lower than that of juvenile plants. Therefore, the change of water use strategy might help this shrub to adapt to the semi-arid environment in the Gonghe Basin on Tibet Plateau. We suggest that improved management strategy, such as irrigation during drought, should be used to increase the growth of plantations of juvenile *C. intermedia*.

## Materials and Methods

### Study site

A survey was conducted in the Desertification Combating Experimental Site of the Gonghe Desert Ecosystem Research Station (latitude N 36°16′, longitude E 100°16′ and altitude 2871 m). It is one of the stations in the Chinese Desert Ecosystem Research Network (CDERN) of the State Forestry Administration of P.R. China. The station is constructed by the Chinese Academy of Forestry and the Desertification Combating Station of Qinghai Province, which is located at Gonghe Basin on the northeastern Tibetan plateau. The region is semi-arid with a temperate climate, a mean annual air temperature of 2.4°C and a mean annual precipitation of 246.3 mm, which is concentrated in the summer and early autumn (July – September). The mean annual potential transpiration is 1716.1 mm, the mean annual number of windy days is 50.6 d and the primary wind direction is north-northwest. The mean annual wind speed is 2.7 m s^–1^ and the mean length of the frost-free season is 91 d. The natural vegetation includes shrubs (e.g. *Artemisia ordosica* and *Caragana tibetica*) and grasses (e.g. *Leymus secalinus*, *Orinus kokonorica* and *Stipa krylovii*). The shelter belt inside and outside the oasis is formed by trees (e.g. *Populus cathayana*) and shrubs (e.g. *Caragana intermedia*, *Salix cheilophila*, *Tamarix chinensis* and *Hippophae rhamnoides*).

### Plant species

*Caragana intermedia* Kuang et H. C. Fu is one of the primary shrubs growing on semi-fixed and fixed sandland and on loam in the hilly gully of the Inner Mongolia Autonomous Region, North Shaanxi Province and the Ningxia Hui Autonomous Region [22]. *C. intermedia* is planted as a fine sand-binding and afforestation shrub growing on sand dunes 3 km to the south of the Gonghe Station. The main type of soil on sand dunes is sandy loam, and clay exists at different soil depths. In August 2009, three different ages (5, 9 and 25 years, planted separately in 2004, 2000 and 1984) of *C. intermedia* plantations were chosen to study the effects of plantation age on the main water source and WUE. Before plant sampling, the height and crown diameter of 20 plants was measured separately in three ages of *C. intermedi*a plantations. The community characteristics of three ages of *C. intermedia* are given in Table 2. A few *Leymus secalinus* (wild rye) grew in all three *C. intermedia* plantations. Moreover, a few *Suadeda glauca* and *Carex* spp. grew in the 9 and 25 years old *C. intermedia* plantations.

### Survey method

The mean value of height and crown diameter of *C. intermedi*a was defined as standard plant. Then four plants that was similar to standard plant were chosen for twig and leaf sample. Samples of soil as well as twigs and leaves of four *C. intermedia* plants were collected in 11–13 August 2009 in all three plantations. Lignified twigs (5 cm long) were collected from the sunny side of five shrubs, the bark was removed and samples of the xylem were placed into glass sample vials (8 mL, National Scientific Company, USA), sealed with Parafilm® (Alcan Packaging, WI, USA) and stored in a medical cool-box at <15°C. Four replicates of soil samples were taken at different depths (10, 20, 30, 50, 100 and 150 cm) using a soil auger (AMS Company, USA) in three ages of *C. intermedia* plantations. The maximal soil survey depth was determined on the basis of soil texture and root distribution of *C. intermedia* plantations of different ages; 100 cm, 150 cm and 150 cm for 5, 9 and 25 years old *C. intermdiea* plantations, respectively. Soil samples were placed into 8 mL glass sample vials, sealed with Parafilm® and stored into a medical cool-box at <15°C. Four replicates of each soil sample were placed into aluminum boxes and used to measure the water content. The wet soil in the aluminum boxes was weighed on an electronic balance (±0.01 g), dried at 105°C for 24 h, then weighed again and the soil water content (g kg^–1^) was calculated as the loss of mass. Water in the xylem and soil samples was vacuum-extracted and the δD/δ^18^O ratio was measured with a Finnigan MAT Delta V advantage mass spectrometer in the Stable Isotope Ratio Mass Spectrometer Laboratory of the Chinese Academy of Forestry (SIRMSL, CAF). On rainy days, rainwater was collected at Gonghe Station before soil and plant sampling. Well water were collected from a 5 m deep well within shelter belt systems, which was used to replace ground water. Three replicate water samples were collected and placed into 8 mL glass sample vials, sealed with Parafilm® and stored in a medical cool-box at <15°C. The δD/δ^18^O ratio of the rainwater and well water was measured by mass spectrometry in the laboratory.

Three replicates of mature leaf samples were collected randomly from the sunny side of five *C. intermedia* plants of the three different ages near the soil sampling sites, placed into paper bags, dried at 105°C for 1 h and then at 80°C for 24 h. Dry leaves were pulverized and passed through an 80-mesh sieve. The δ^13^C ratio of the leaves was measured by mass spectrometry in the laboratory. The leaf δ^13^C ratio was converted to carbon isotope discrimination (Δ, ‰) ratios, using an atmospheric carbon dioxide ratio of –8‰ [23].

A root survey was done in August 20, 2010. Three replicates of soil profile (50 cm ×50 cm) was dug by shovel in each age of *C. intermedia* plantations. The maximal depth of root survey was 100 cm for the 5 years old plantation and 130 cm for the 9 and 25 years old plantations. Soil was collected at every 10 cm deep and passed through a 2 mm mesh sieve. The *C. intermedia* roots were divided into two groups according to diameter (fine root, ≤1 mm; prop root, >1 mm), dried at 75°C for 24 h, weighed on an electronic balance (accurate to ±0.1 g) and root dry mass (g) was transformed into root density (g m^–3^).

### Data analysis

Soil water content, δD and δ^18^O ratio, and carbon discrimination are expressed as mean ± SE. Water use ratio of different sources was analyzed by Iso-source 1.3.1 software (downloaded from http://www.epa.gov/wed/pages/models/stableIsotopes/isosource/isosource.htm) for the three *C. intermedia* plantations of different ages. The results of water use ratio are expressed as mean ± SD. One-way analysis of variance (ANOVA) to compare the effects of plantation age on different indices was done with SPSS 17.0 software. If the effect was statistically significant (*p*<0.05), Duncan's multiple-range test was used to compare the difference between plantation ages.

## References

[1] Ehleringer JR (1993) Carbon and water relations in desert plants: an isotopic perspective. In: Ehleringer JR, Hall AE, Farquhar GD, editors. Stable Isotope and Plant Carbon-Water Relations. San Diego: Academic Press. 155–172.

[2] Dodd MB, Lauenroth WK, Welker JM (1998) Differential water resource use by herbaceous and woody plant life forms in a short grass steppe community. Oecologia 117: 504–512. 28307675 10.1007/s004420050686

[3] Gebauer RLE, Ehleringer JR (2000) Water and nitrogen uptake patterns following moisture pulses in a cold desert community. Ecology 81: 1415–1424.

[4] Schwinning S, Davis K, Richardson L, Ehleringer JR (2002) Deuterium enriched irrigation indicates different forms of rain use in shrub/grass species of the Colorado Plateau. Oecologia 130: 345–355. 28547040 10.1007/s00442-001-0817-0

[5] Schwinning S, Starr BI, Ehleringer JR (2003) Dominant cold desert plants do not partition warm season precipitation by event size. Oecologia 136: 252–260. 12695904 10.1007/s00442-003-1255-y

[6] Chimner RA, Cooper DJ (2004) Using stable oxygen isotopes to qualify the water source utilized for transpiration by native shrubs in San Luis Valley, Colorado U.S.A. Plant and Soil. 206: 225–236.

[7] Schwinning S, Starr BI, Ehleringer JR (2005) Summer and winter drought in a cold desert ecosystem (Colorado Plateau) part I: effects on soil water and plant water uptake. Journal of Arid Environments 60: 547–566.

[8] Li G, Romero-Saltos H, Tsujimura M, Sugimoto A, Sasaki L, et al (2007) Plant water sources in the cold semiarid ecosystem of the upper Kherlen River catchment in Mongolia: A stable isotope approach. Journal of Hydrology 333: 109–117.

[9] Ohte N, Koba K, Yoshikawa K, Sugimoto A, Matsuo N, et al (2003) Water utilization of trees in semiarid desert of Inner Mongolia, China. Ecological Applications 13 (2): 337–351.

[10] Cheng X, An S, Li B, Chen J, Lin GH, et al (2006) Summer rain pulse size and rainwater uptake by three dominant desert plants in a desertified grassland ecosystem in Northwest China. Plant Ecology 184: 1–12.

[11] Yang H, Auerswald K, Bai Y, Han X (2010) Complementarity in water sources among dominant species in typical steppe ecosystems of Inner Mongolia, China. Plant and Soil 340: 141–155.

[12] Zhu Y, Jia Z, Lu Q, Hao Y, Zhang J, et al (2010) Water use strategy of five shrubs in Ulanbuh Desert. Scientia Silvae Sinica 46 (4): 15–21 (in Chinese with English abstract)..

[13] Zhu Y, Jia Z, Yang X (2011) Resource-dependent water use efficiency of two desert shrubs on interdune, Northwest China. Journal of Food, Agriculture & Environment 9: 832–835.

[14] Zhu Y, Jia Z (2011) Soil water utilization characteristics of *Haloxylon ammodendron* plantation with different age during summer. Acta Ecologica Sinica 31: 341–346.

[15] Dawson TE, Mambelli S, Plamboeck AH, Templer PH, Tu KP (2002) Stable isotopes in plant ecology. Annual Review of Ecology and Systematics 33: 507–559.

[16] Donovan LA, Ehleringer JT (1994) Carbon isotope discrimination, water-use efficiency, growth and mortality in a natural shrub population. Oecologia 100: 347–354. 28307020 10.1007/BF00316964

[17] Li S, Dong Y, Dong G (2001) Desertification and Sustainable Development on Tibet Plateau. Beijing: Chinese Tibetlogy Press. 253–269. (in Chinese).

[18] Darrouzet-Nardi A, D'Antonio M, Dawson TE (2006) Depth of water acquisition by invading shrubs and resident herbs in a Seirra Nevada meadow. Plant and Soil 285: 31–43.

[19] Cheng X, Huang M, Shao M, Warrington DN (2009) A comparison of fine root distribution and water consumption of mature *Caragana korshinskii* Kom. grown in two soils in a semiarid region, China. Plant and Soil 315: 149–161.

[20] Casper BB, Forseth IN, Wait DA (2005) Variation in carbon discrimination in relation to plant performance in a natural population of *Cryptantha flava* . Oecologia 145: 541–548. 16010535 10.1007/s00442-005-0162-9

[21] Zhao L, Xiao H, Liu X (2006) Variations of folia carbon isotope discrimination and nutrient concentrations in *Artemisia ordosica* and *Caragana korshiskii* at the southeastern margin of China's Tengger Desert. Environmental Geology 50: 285–294.

[22] Fu K (1992) Flora Republicae Popularis Sinicae 42 (1): Leguminosae. Beijing: Science Press. 47–49. (in Chinese).

[23] West JB, Bowen GJ, Cerling TE, Ehleringer JR (2006) Stable isotopes as one of nature's ecological records. Trends in Ecology and Evolution 21: 408–414. 16753238 10.1016/j.tree.2006.04.002

